# Emerging threat of Oropouche virus in Brazil: an urgent call for enhanced surveillance and response

**DOI:** 10.1016/j.bjid.2024.103876

**Published:** 2024-09-30

**Authors:** Mateus Santana do Rosário, Isadora Cristina de Siqueira

**Affiliations:** a Ministério da Saúde, Fundação Oswaldo Cruz, Instituto Gonçalo Moniz, Laboratório de Investigação em Saúde Global e Doenças Negligenciadas, Salvador, BA, Brazil; b Universidade do Estado da Bahia, Departamento de Ciências da Vida, Campus I, Salvador, BA, Brazil

Dear Editor,

Oropouche Virus (OROV) is an arbovirus belonging to the Orthobunyavirus genus, typically transmitted by female Culicoides paraensis midges.^1^ The first isolation of the virus was in 1955 in Trinidad and Tobago.^2^ Since then, it has been responsible for several outbreaks in Central and South America, particularly in the Amazon region.^1^

In Brazil, the first outbreak of Oropouche fever was described in 1961 in Belém, with 11,000 cases reported.^3^ Since then, several self-limited outbreaks of OROV or sporadic cases have been reported in Amazon regions.^4^ More recently, OROV has been identified in other regions in Brazil, highlighting the potential for the spread of the virus in non-endemic areas.^5^^,^^6^

In 2023, OROV molecular diagnosis was decentralized to the country's Central Public Health Laboratories (LACEN), with 831 samples positive for OROV by RT-PCR identified. In 2024, an increase in the number of detected cases was reported, with 6976 positive samples up to epidemiological week 26. Most cases have been reported in the Amazon region. However, positive samples were also detected in the states of Bahia, Pernambuco, Piauí, Maranhão, Espírito Santo, Minas Gerais, Rio de Janeiro, Mato Grosso, and Santa Catarina.^7^

The disease typically presents with headache, arthralgia, myalgia, nausea, vomiting, chills, and photophobia.^8^^,^^9^ The overlapping symptomatology with other arboviral infections imposes a challenge on the diagnosis and management, potentially leading to misdiagnosis.^9^

Although OROV infections are typically self-limiting, a few severe cases have been reported, such as hemorrhagic abnormalities, meningitis, and encephalitis, which necessitate hospitalization and intensive medical intervention.^8^^,^^10^ Despite the description of epidemics since the 1960s, no fatal case of OROV infection had been described until June 2024, when the Bahia Health Department confirmed the association of two deaths with the OROV infection.^11^

Furthermore, the recent identification of two possible cases of vertical transmission of OROV is a significant concern. A pregnant woman from Pernambuco presented with febrile illness in 30ª gestational weeks that evolved to fetal death a few days later. The OROV genetic material was detected in Placenta, umbilical cord blood, and fetus organ tissues. The second reported case was a pregnant woman in the 6ª gestational week who presented with acute OROV infection confirmed by RT-PCR in a blood sample. She evolved with uterine hemorrhage and miscarriage.^12^

Considering the prevailing trends and the socio-environmental factors, OROV outbreaks will likely continue to increase in Brazil.^8^ To mitigate the impact of this virus, it is imperative to strengthen vector control strategies, improve diagnostic capabilities, and increase public awareness of preventive measures.^8^ Collaboration between healthcare providers, researchers, and policymakers is essential to address this emerging threat. Strengthening surveillance, improving diagnostic accuracy, and implementing effective public health interventions will facilitate more effective management and mitigation of the impact of OROV on affected populations.

## Conflicts of interest

The authors declare no conflicts of interest.
